# The importance of perinatal maternal depression as a public health problem in Africa

**DOI:** 10.1016/S2215-0366(22)00197-3

**Published:** 2022-05-19

**Authors:** Symon M Kariuki, Charles RJC Newton

**Affiliations:** Neuroscience Unit, KEMRI–Wellcome Trust Research Programme, Kilifi 80108, Kenya; Department of Public Health, Pwani University, Kilifi, Kenya; Department of Psychiatry, University of Oxford, Oxford, UK; Neuroscience Unit, KEMRI–Wellcome Trust Research Programme, Kilifi 80108, Kenya; Department of Public Health, Pwani University, Kilifi, Kenya; Department of Psychiatry, University of Oxford, Oxford, UK

The prevalence of perinatal maternal depression in low-income and middle-income countries (LMICs) is estimated to be 17–30%^1–3^ higher than in high-income countries. There were relatively few African studies (eg, from Kenya) in these systematic reviews^2,3^ and there is considerable heterogeneity because of differences in sample sizes, study design, and assessment scales (often with no clear cross-cultural adaption of these scales). The predisposing factors for maternal depression, including childhood trauma, low socioeconomic status, and intimate partner violence and psychosocial stress during pregnancy, are more common in LMICs than in high-income countries. The psychosocial model proposed by Bronwyn Leigh and Jeannette Milgrom^4^ added personal resources (eg, low self-esteem and negative cognitive style) to the risk factors; but these are rarely explored in studies of perinatal maternal depression. Perinatal maternal depression affects not only the health of the mother, but also that of the child through their relationship and interaction (eg, breastfeeding and bonding).^5^ One of the key targets for UN Sustainable Development Goal 3 is universal access to sexual and reproductive health-care services, achievement of which can be accelerated through development of perinatal mental health policies and generation of empirical evidence to track progress and to inform planning of care.

The study published in *The Lancet Psychiatry* by Anna Larsen and colleagues^6^ is timely and provides important data on the extent and trajectories of perinatal maternal depression from a sub-Saharan Africa country, Kenya, where there is scarce empirical evidence from such well powered studies. The findings from this study confirm the high prevalence of perinatal maternal depression and the known risk factors (eg, limited social support and partner with HIV or partner with unknown HIV status). Larsen and colleagues’ study^6^ provides novel insights into the characterisation of resolving, persisting, and emerging antepartum and post-partum depressive symptoms using group-based trajectory modelling (a method that groups individuals with similar patterns of an outcome measured over time using maximum likelihood estimation). These identified trajectories of psychopathology have public health importance; for example, the need to institute early interventions for persisting symptoms, and continuous screening and treatment for post-partum emergence of symptoms. The findings from Larsen and colleagues’ study suggest that at least a fifth of cases of perinatal maternal depression could be alleviated through social support targeted at women with intimate partner violence and previous pregnancy loss. Perinatal women would be likely to benefit from a range of psychosocial and psychiatric interventions to prevent and treat perinatal maternal depression.

Larsen and colleagues admit key limitations of their study (eg, an absence of clinical diagnosis and confounding by unmeasured factors), some of which could be addressed in future studies. For example, the role of the general health status of the mothers was not investigated, and neither was the role of culture in perinatal maternal depression (eg, stress from sex expectations of the unborn child, whereby male babies might be more wanted than female babies). The role of climate and environment on perinatal maternal depression is unclear, yet exposure to heat stress during pregnancy can contribute to poor maternal and child outcomes. The timing of depression during pregnancy might be important, but few studies document the trimesters when depressive symptoms start. Partner psychopathology might be important in perinatal maternal depression, but was not explored in Larsen and colleagues’ study; the analysis did include partner HIV-positive status, which is crucial in Africa. Although poor neurodevelopment of children might follow maternal depression, this was not assessed in Larsen and colleagues’ study. Future studies require a comprehensive assessment of varied outcomes to understand the complexity of the factors and their inter-relationship with the development and consequences of perinatal maternal depression. Understanding the effect of perinatal maternal depression on parenting is a crucial mechanism for preventing the intergenerational transmission of poor health outcomes to offspring.^5^ Long-term follow-up beyond the 9 months post partum, as in the present study, is important to understand the course of perinatal maternal depression and its effect on health across the lifespan of the mother and child.^5^ Mechanisms underlying these poor health outcomes were not characterised in Larsen and colleagues’ study, yet neurobiological stress response systems, such as the hypothalamic–pituitary–adrenal axis and the inflammatory system, as well as alterations in brain function and structure, are perturbed even in healthy pregnancy and can mediate poor outcomes following childhood trauma in the presence of risk factors.^7^

Future research efforts should focus on identification of depression during the antenatal and postnatal periods, with appropriate tool adaptation and validation, and integration of mental health care into maternal and child health services. Mental health assessment, treatment, and preventive services can be offered during antenatal care visits. Encouraging antenatal visits might reduce risk of pregnancy complications or losses, which are antecedents for perinatal maternal depression. Future studies should examine the long-term effect on various health outcomes of the child and mother and the value of perinatal mental health assessment scales in predicting these outcomes. Specific interventions might include supportive and behavioural interventions for expectant mothers, socioeconomic and education empowerment, prevention of risk factors for perinatal maternal depression, strengthening of mental health systems, and treatment of mental illnesses experienced outside of pregnancy. Maternal–child mental health is an important public health issue^8^ because it affects the mother’s and child’s health throughout the lifespan, curtailing human capital and economic development. Importantly, existing and new legislation and laws should recognise perinatal maternal depression as an important public health problem and support mandatory screening of depression during pregnancy.^7^

## References

[1] Gelaye B, Rondon MB, Araya R, Williams MA (2016). Epidemiology of maternal depression, risk factors, and child outcomes in low-income and middleincome countries. Lancet Psychiatry.

[2] Dadi AF, Wolde HF, Baraki AG, Akalu TY (2020). Epidemiology of antenatal depression in Africa: a systematic review and meta-analysis. BMC Pregnancy Childbirth.

[3] Dadi AF, Akalu TY, Baraki AG, Wolde HF (2020). Epidemiology of postnatal depression and its associated factors in Africa: a systematic review and meta-analysis. PLoS One.

[4] Leigh B, Milgrom J (2008). Risk factors for antenatal depression, postnatal depression and parenting stress. BMC Psychiatry.

[5] Slomian J, Honvo G, Emonts P, Reginster J-Y, Bruyère O (2019). Consequences of maternal postpartum depression: a systematic review of maternal and infant outcomes. Womens Health.

[6] Larsen A, Pintye J, Mary MM (2022). Trajectories and predictors of perinatal depressive symptoms among Kenyan women: a prospective cohort study. Lancet Psychiatry.

[7] Biaggi A, Conroy S, Pawlby S, Pariante CM (2016). Identifying the women at risk of antenatal anxiety and depression: a systematic review. J Affect Disord.

[8] Meaney MJ (2018). Perinatal maternal depressive symptoms as an issue for population health. Am J Psychiatry.

